# Supplementary material for: To Trim or Not to Trim: Effects of Read Trimming on the De Novo Genome Assembly of a Widespread East Asian Passerine, the Rufous-Capped Babbler (Cyanoderma ruficeps Blyth)
Source: Genes (Basel). 2019 Sep 23;10(10):737. doi: 10.3390/genes10100737 (PMC6826712; doi:10.3390/genes10100737)
Supplement: Supplementary file 1 [file genes-10-00737-s001.pdf]

# To trim, or not to trim: effects of read-trimming on the *de novo* genome assembly of a widespread East Asian passerine, the rufous-capped babbler (*Cyanoderma ruficepes*)

Shang-Fang Yang[1]†, Chia-Wei Lu[1]†, Cheng-Te Yao[2] and Chih-Ming Hung[1]

[1] Biodiversity Research Center, Academia Sinica, Taipei, Taiwan

[2] Division of Zoology, Endemic Species Research Institute, Nantou, Taiwan

## Supplementary Figures: Figs. S1 and S2

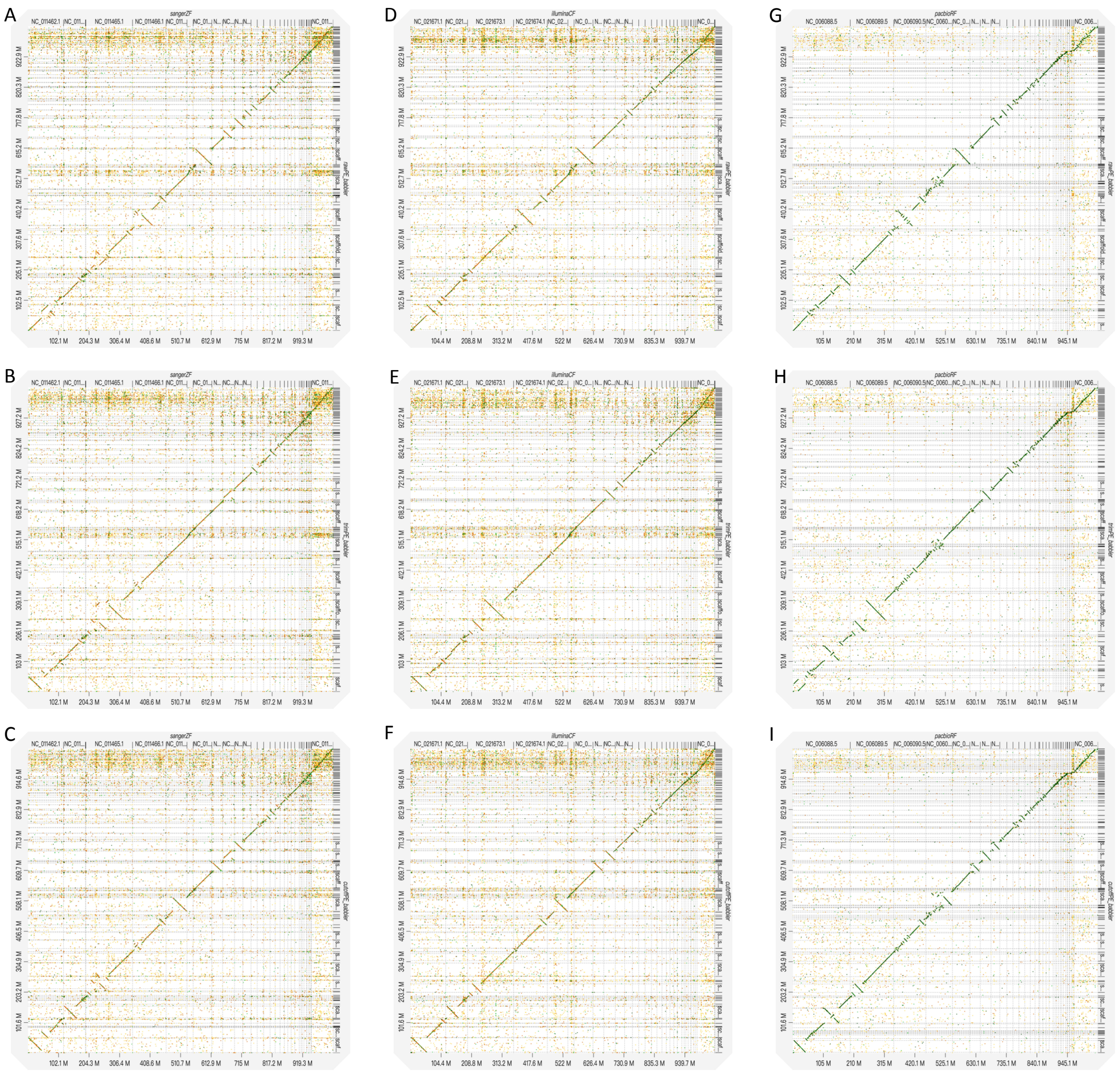

**Figure S1.** D-GENIES plots of alignments between the rufous-capped babbler and three avian genomes. The left panels show that the raw PE (A), trimmed (B), and cut off (C) PLATANUS-assembled genomes mapped to the zebra finch genome. The middle panels show that the raw PE (D), trimmed (E), and cut off (F) PLATANUS-assembled genome mapped to the collar flycatcher genome. The right panels show that the raw PE (G), trimmed (H), and cut off (I) PLATANUS-assembled genome are mapped to the chicken genome. Only the scaffolds of the rufous-capped babbler genomes with lengths > 5,000 bp were used. The unlocalized scaffolds of the three reference genomes were excluded from analyses.

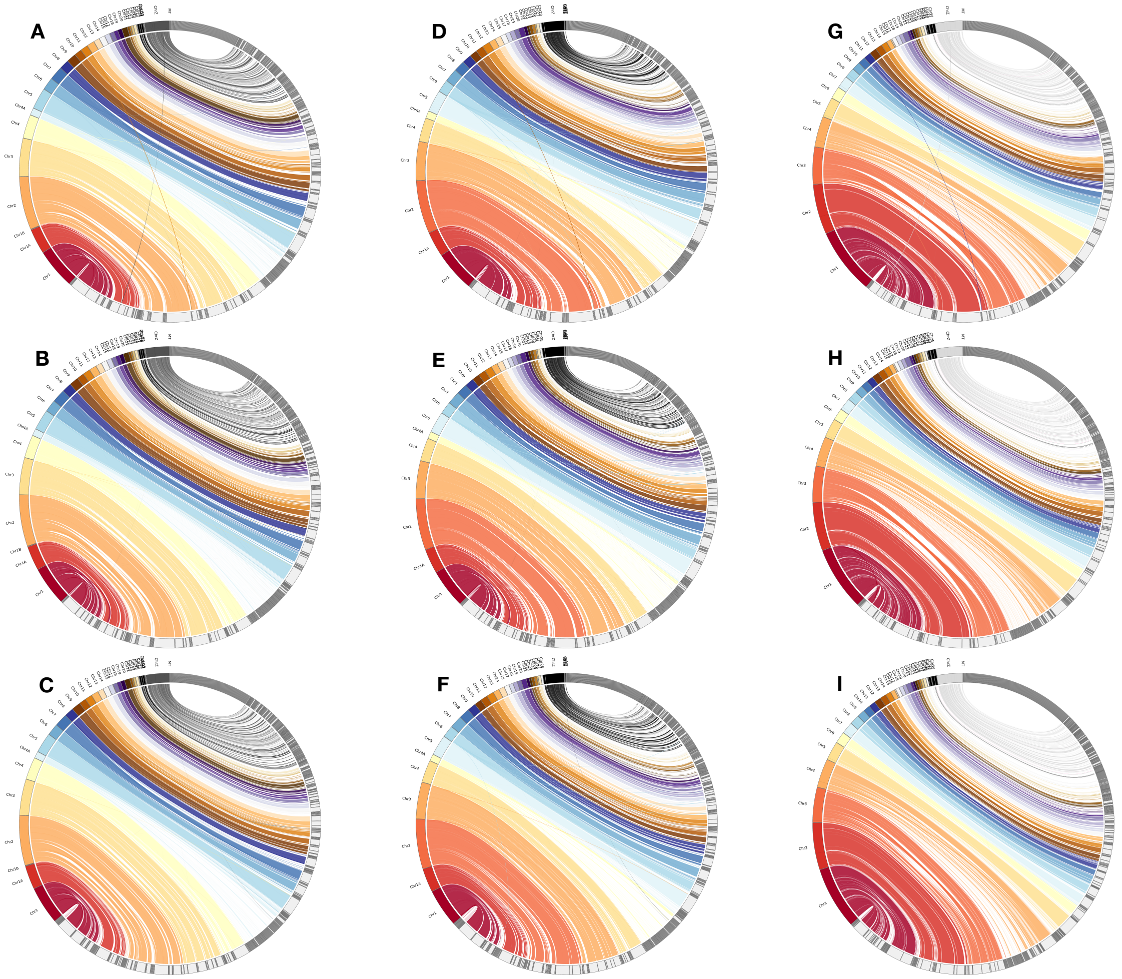


**Figure S2.** Jupiter plots of alignments between the rufous-capped babbler and three avian genomes. The left panels show that the raw PE (A), trimmed (B), and cut off (C) PLATANUS-assembled genomes mapped to the zebra finch genome. The middle panels show that the raw PE (D), trimmed (E), and cut off (F) PLATANUS-assembled genome mapped to the collar flycatcher genome. The right panels show that the raw PE (G), trimmed (H), and cut off (I) PLATANUS-assembled genome are mapped to the chicken genome. Only the scaffolds of the rufous-capped babbler genomes with lengths > 5,000 bp were used. The unlocalized scaffolds of the three reference genomes were excluded from analyses.
